# Strong Electro‐Optic Effect and Spontaneous Domain Formation in Self‐Assembled Peptide Structures

**DOI:** 10.1002/advs.201700052

**Published:** 2017-05-11

**Authors:** Barak Gilboa, Clément Lafargue, Amir Handelman, Linda J. W. Shimon, Gil Rosenman, Joseph Zyss, Tal Ellenbogen

**Affiliations:** ^1^ Department of Physical Electronics Fleischman Faculty of Engineering Tel‐Aviv University Tel‐Aviv 69978 Israel; ^2^ Laboratoire de Photonique Quantique et Moléculaire (LPQM UMR CNRS 8537) Ecole Normale Supérieure de Cachan Université Paris‐Saclay 67 Avenue du Président Wilson 94235 Cachan France; ^3^ Faculty of Engineering Department of Electrical Engineering Holon Institute of Technology (HIT) 52 Golumb St. Holon 5810201 Israel; ^4^ Department of Chemical Research Support Weizmann Institute of Science Rehovot 76100 Israel; ^5^ Center for Light‐Matter Interaction Tel‐Aviv University Tel‐Aviv 6779801 Israel

**Keywords:** domain formation, guest molecules, peptide structures, Pockels effect, self‐assembly

## Abstract

Short peptides made from repeating units of phenylalanine self‐assemble into a remarkable variety of micro‐ and nanostructures including tubes, tapes, spheres, and fibrils. These bio‐organic structures are found to possess striking mechanical, electrical, and optical properties, which are rarely seen in organic materials, and are therefore shown useful for diverse applications including regenerative medicine, targeted drug delivery, and biocompatible fluorescent probes. Consequently, finding new optical properties in these materials can significantly advance their practical use, for example, by allowing new ways to visualize, manipulate, and utilize them in new, in vivo, sensing applications. Here, by leveraging a unique electro‐optic phase microscopy technique, combined with traditional structural analysis, it is measured in di‐ and triphenylalanine peptide structures a surprisingly large electro‐optic response of the same order as the best performing inorganic crystals. In addition, spontaneous domain formation is observed in triphenylalanine tapes, and the origin of their electro‐optic activity is unveiled to be related to a porous triclinic structure, with extensive antiparallel beta‐sheet arrangement. The strong electro‐optic response of these porous peptide structures with the capability of hosting guest molecules opens the door to create new biocompatible, environmental friendly functional materials for electro‐optic applications, including biomedical imaging, sensing, and optical manipulation.

## 1 Introduction

Self‐assembled peptide structures have been researched extensively in recent years due to their potential in numerous important biocompatible applications.^1^, ^2^, ^3^, ^4^, ^5^ A privileged position among self‐assembling peptides is reserved for short phenylalanine repeats, owing to the diversity of self‐assembled structures that they display including tubes, tapes, spheres, and fibrils.^6^, ^7^, ^8^, ^9^, ^10^ This structural diversity has found potential applications in different fields including scaffolds for regenerative medicine, light harvesting materials, fluorescent probes, and mechanically tunable hydrogels, to name but a few.^11^, ^12^, ^13^, ^14^, ^15^ The interest in these ultrashort peptides originated from the extensive research on amyloid fibrils, which are self‐assembled fibrillar structures formed by misfolding peptides or proteins, that were shown to be involved in several degenerative diseases including Alzheimer's, Parkinson's, type II diabetes and more.^16^, ^17^, ^18^, ^19^, ^20^, ^21^ In particular, in 2002, Gazit showed that the dipeptide diphenylalanine (FF) is the core recognition motif of the beta amyloid polypeptide implicated in Alzheimer's disease.^22^ FF was found to self‐assemble into nanoscaled tubes in water with remarkable physical properties such as large mechanical stiffness, super hydrophobicity, supercapacitance, and strong piezoelectric response.^23^, ^24^, ^25^, ^26^, ^27^ The latter property is associated with a lack of inversion symmetry of the material's structure, which is also associated with quadratic optical nonlinearity, enabling optical frequency conversion. This was very recently confirmed by Handelman et al. that demonstrated efficient second harmonic generation (SHG) from FF tubes and triphenylalanine (FFF) nanotapes.^9^

One of the most basic and widely applicative physical phenomenon related to noncentrosymmetric structures is the linear electro‐optic effect.^28^ Materials possessing this property change their refractive index linearly as a response to an externally applied electric field.^29^ This effect is used to implement a variety of functional optical components including fast optical switches, amplitude modulators, Q‐switching, pulse pickers, and fast phase modulators.^30^ The ability to use electro‐optic active peptide structures can open the door to new types of electro‐optical biocompatible probes, biomedical sensors, and even flexible electro‐optic devices.

Here we measure for the first time the effective electro‐optic coefficients of self‐assembled FF peptide tubes (FF‐tubes) and FFF peptide tapes (FFF‐tapes) and find that they have remarkably large values, with FF‐tubes values comparable to that of the best inorganic crystals. In addition we measured the X‐ray diffraction (XRD) pattern of FFF‐tapes, which shows a nanoporous structure and extensive antiparallel β‐sheet arrangement. Moreover, we also observed the spontaneous formation of domains which can potentially be controlled to form nonlinear photonic crystals in the peptide structures.^31^ These findings form the foundations to use peptides in electro‐optic applications.

## 2 Results and Discussion

**Figure 1:**
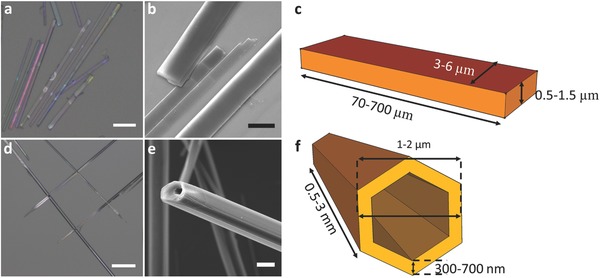
The structure of FF and FFF supramolecular assemblies. a–c) The structure of FFF. a) Reflection bright field image. Bar size is 50 µm. b) SEM image of FFF‐tapes. Bar size is 5 µm. c) Illustration of FFF‐tape and its typical dimensions. d–f) The structure of FF. d) Reflection bright field image. Bar size is 25 µm. e) SEM image of FF‐tube. Bar size is 5 µm. f) Illustration of FF‐tube and its typical dimensions.

We examined two types of polypeptide structures, FFF‐tapes and FF‐tubes (**Figure** **1**). The FFF‐tapes grow into elongated structures with rectangular cross sections. Their thicknesses range from a few hundreds of nanometers and up to ≈1.5 µm. Their widths usually range between 3 and 6 µm and they are up to hundreds of micrometers in length (Figure 1c). The FF‐tubes that we grew (see the Experimental Section) self‐assemble in water into tubes with hexagonal cross sections (Figure 1d–f). Their typical wall thickness is of a few hundreds of nanometers, diameters of 1–2 µm typically, and lengths that can extend up to millimeters (see also Figure S1, Supporting Information).

**Figure 2:**
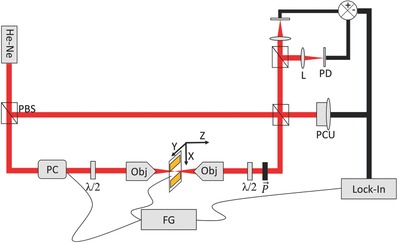
The Pockels linear electro‐optic microscope setup. Major components of PLEOM: He–Ne, helium–neon laser; PBS, polarizing beam splitter; λ/2, half wave plate; PC, Pockels cell; Obj, objective; gold bars, electrodes; FG, function generator; $\vec{P}$, polarizer; PCU, phase correcting unit; L, lens; PD, photodiode; XYZ, defines the axes in the laboratory frame.

In order to determine the electro‐optic response of the FFF‐tapes and FF‐tubes, we imaged the structures using a custom built Pockels linear electro‐optic microscope (PLEOM).^32^ PLEOM is a Mach–Zender type interferometric microscope whose sample arm passes through a confocal optical configuration, where an alternating electric field is applied to two electrodes placed in the focal plane a few µm a part (**Figure** **2**, see the Experimental Section for a detailed description of operation).

In electro‐optic active materials, an applied electric field modifies the refractive index of the material. Assuming a small change in the refractive index in response to the applied electric field, the index change, Δ*n*, can be accounted for as follows

(1) $$\Delta n_{i}≅\frac{1}{2}n_{i}^{3}\sum_{j}r_{ij}\cdot E_{j}$$

where *i* stands for a couple of dielectric axes according to the usual convention,^33^ *n_i_* are the unperturbed respective refractive indices, *r_ij_*  are the electro‐optic tensor elements, and *E_j_* is the applied electric field along the *j*th axis. Therefore, a beam that passes through the focal plane will experience a phase retardance

(2) $$\Delta \varphi_{i}=\frac{2\pi }{\lambda }\Delta n_{i}d$$

where λ is the wavelength of incident light and *d* is the distance that light travels in the index modulated material. In the PLEOM, the modulated beam is then recombined with a reference beam and projected onto two photodiodes in balanced homodyne configuration, whose signal is subsequently fed to a lock‐in amplifier. The amplitude signal in the amplifier is directly proportional to the retarded phase of the sample arm,^34^ while the measured phase in the lock‐in amplifier, Θ, is related to the polarization direction in the material.^35^ This setup allows for a direct measurement of the electro‐optic effect in the studied material with very high precision, down to a sensitivity of 10^−6^ radians in phase variation.

### 2.1 Electro‐Optic Imaging of FFF‐Tapes

**Figure 3:**
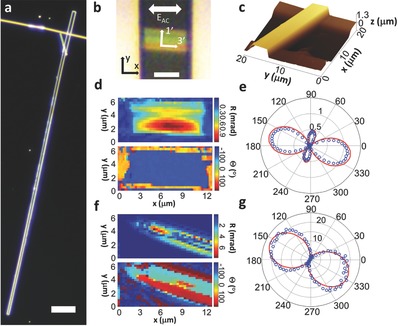
Electro‐optic characterization of FFF‐tape and FF‐tube. a) Dark field image of a long tape. Bar size is 30 µm. b) Bright field image of the tape analyzed in (d) and (e). *E*_ac_ denotes the direction of electric field. *x*–*y* define the laboratory framework. 1′–3′ define the crystal dielectric axes. Bar size is 5 µm. c) An AFM image of a typical tape on top of the electrodes. d) Retardance and Θ images obtained concurrently by PLEOM of the tape in (b). e) Polar plot of the tape imaged in (d). Angles are defined according to the laboratory framework. Values are in mrad. Red line is a fit to the data. f) Retardance and Θ images obtained concurrently by PLEOM for a FF‐tube. g) Polar plot for the tube in (f). Values are in mrad. Red line is a fit to the data.

**Figure** **3**a shows a typical long FFF‐tape of several hundreds of micrometers that was grown. In order to image the tapes, a tapes containing drop was taken from the aged solution and gently placed on top of a gold electrodes array fabricated on a microscope cover slip (see the Experimental Section and Figure S2, Supporting Information). The process resulted in many tapes placed on top of the electrodes with random orientations.

Figure 3b shows a closeup of another FFF‐tape with an orientation of ≈5° between the long axis (marked as 3′) of the FFF‐tape and the applied electric field by the electrodes, *E*_AC_. A scanning PLEOM image of the retardance (R in Figure 3 and henceforth) for this FFF‐tape orientation is shown in Figure 3d. This image was taken with light polarized in a 5° angle to the *x*‐direction of the laboratory frame which is parallel to the applied field (Figure 3b). Despite the low thickness of the crystal, the response was surprisingly strong and indicative of high values for the electro‐optic coefficients. The peak signal is located slightly below the center of the FFF‐tape along the *x*‐axis due to slight misalignment of the slide with respect to the laser light, which was also observed for other similarly aligned tapes. The response was linear over the range of the applied electric field, which indicates that it originates from the linear electro‐optic effect and that the applied field within this range does not change the dipolar structure nor saturates the response (Figure S3, Supporting Information). The scanning image of the Θ signal of PLEOM (Figure 3d) was relatively uniform across the tape, only changing at the left and right sides where the electrodes reside, and the signal becomes severely attenuated (observed also in the retardance image). This constant Θ value across the entire FFF‐tape measurement area suggests a single domain structure due to the sensitivity of the phase to the orientation of the molecular dipoles in the peptide crystal. In order to shed light on the dipole orientation in the FFF‐tape, we measured the polarimetric response of the crystal by changing the incident polarization of the light on the sample (Figure 3e). The plot shows a predominant response in the direction of the long axis of the tube (3′), and a weaker response perpendicular to it (1′). Our result suggests for this FFF‐tape an orientation of the dipoles along the tape, similar to the results obtained for the SHG from FFF nanotapes^9^ and for FF‐tubes.^9^, ^26^, ^36^ This result repeated itself for other FFF‐tapes with differing orientation with regards to the applied field (Figure S4, Supporting Information), indicating it is the typical response for these crystals. The results did not show any change in the shape of the polarization plot as a function of the sample thickness, and the signal increased with thickness as expected from the Pockels effect.

Polarization plots allow us to calculate the linear electro‐optic coefficients along the tape (3′) and perpendicular to it (1′) as defined above (Figure 3b). However, from Equation (1) it is clear that a full determination of the electro‐optic coefficients also requires knowledge of the sample thickness and its refractive indices. We therefore utilized an atomic force microscope (AFM) to measure the thickness of the tapes in our sample (Figure 3c). The AFM images revealed a rather smooth surface for the tapes, with a thickness range between 0.8 and 1.5 µm, and profile roughness, *R*_a_, estimated as the average absolute deviation from the mean, of just 2.6 nm.

In order to determine the refractive indices of our crystals, polarization dependent interferometric phase microscopy of FFF‐tapes was carried out in a τ‐interferometer equipped custom microscope (see the Experimental Section).^37^ The resultant optical path difference of the orthogonal axes, together with the known thickness of the tape allows us to determine the refractive indices of the crystal. The crystal exhibited a refractive index of 1.6 along the tape and 1.55 perpendicular to it. This result is very similar to a value of ≈1.6 for the refractive index of l‐phenylalanine.^38^, ^39^

In order to translate the measured lock‐in amplitude to actual phase retardance, we used a commercial Pockels cell with a known retardance, placed in the sample arm (Figure 2), and measured the signal received by the amplifier. From the measurements we calculated a coefficient of ≈3.8 pm V^−1^ along the tape (*r*′_33_), and ≈1.4 pm V^−1^ perpendicular to it (*r*′_13_). This result is similar to equivalent coefficients of commercially available inorganic electro‐optic crystals such as Barium borate (BBO).^30^

### 2.2 Electro‐Optic Imaging of FF‐Tubes

We have also used the PLEOM to measure the response of FF‐tubes (Figure 3f,g). The complex geometry of the tube manifests itself both in the amplitude and Θ‐images we obtained. The higher thickness of the periphery compared to the center of the tube contributes to a higher signal observed at the edges of the tube, while considerably lower values are seen in the center (Figure 3f). The Θ‐image also shows different values between the periphery and the center in further support of our conclusion. In order to determine the electro‐optic coefficients a polarimetric response was measured which exhibited very high values (Figure 3g). The response was aligned mostly along the long axis of the tube, as was seen before also for SHG from FF.^9^ Using a refractive index of ≈1.6, effective thickness of ≈1.2 µm (see Note S1, Supporting Information), and given FF‐tubes' space group of P6_1_,^40^ we determined values of ≈32 and ≈3.5 pm V^−1^ for the coefficient along the tube (*r*_33_), and perpendicular to it (*r*_13_), respectively (see Note S2, Supporting Information). These results are comparable to the respective coefficients achieved for the best inorganic materials such as lithium niobate (LiNBO_3_) and potassium titanyl phosphate (KTP),^30^ that are transparent in the visible range. It is important to note that since the measurement is done at 60 kHz, part of the high response may be due to the respective piezoelectric coefficients which were very recently measured for FF‐tubes.^41^ The piezoelectric induced strain can cause a significant change in the refractive indices, which adds to the change caused by the Pockels effect.^33^ Therefore, the obtained high values should be regarded as the unclamped electro‐optic coefficients.

### 2.3 FFF‐Tapes Structure

**Figure 4:**
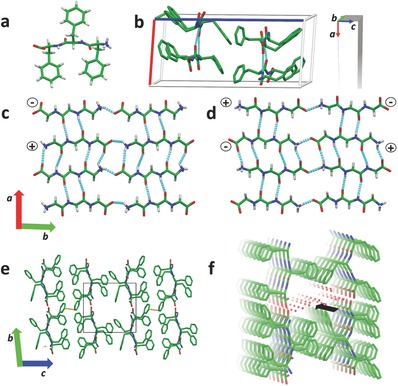
Structural analysis of FFF‐tape. a) FFF zwitterion structure. Nitrogen is blue, Oxygen is red and the carbon backbone is green. b) Structure of the FFF‐tape unit cell. Dashed Cyan lines indicate hydrogen bonds. Hydrogens and waters are omitted for clarity. Unit cell axes color code: *a* is red, *b* is green, and *c* is blue. Inset: Alignment of the unit cell with regards to the supramolecular tape structure. c,d) β‐sheets formed from the four β‐strands in the unit cell along the *a*–*b* plane. The charged end groups are marked accordingly. e) View along the *b*–*c* plane. Dashed orange indicates π–π interactions along the *c*‐axis. Black rectangle highlights the void in the structure. f) Pore channel structure formed by extending the structure along the *a*‐axis. Black arrow is a guide to the eye of the channel axis. Red dots are oxygens within water molecules.

While the structure of FF‐tubes is well known,^40^, ^42^ no structural information is known for the FFF‐tapes that we have grown. Therefore, the crystal structure was determined by single crystal XRD measurements to 1.1 Å resolution in order to elucidate the origin of the electro‐optic response in the FFF‐tapes. The determined structure was found to be triclinic, space group P1, with four FFF molecules and 12 waters per asymmetric unit (**Figure** **4** and Table S1, Supporting Information).

Figure 4a shows the FFF tripeptide in its zwitterionic form, as it appears in the crystal structure, consisting of three phenyl aromatic side chains (green) available for π‐interactions, and two internal amides (nitrogens in blue and oxygens in red) available for hydrogen bonding as well as charged end groups capable of salt bridge formation. The molecules assemble into four antiparallel β‐strands stabilized by hydrogen bonds (dashed cyan lines are hydrogen bonds, Figure 4b). The alignment of the unit cell with regards to the supramolecular tape structure (inset in Figure 4b) reveals the crystallographic *a*‐axis is aligned with the long dimension of the tape, while the *c* crystallographic axis is aligned with the short dimension which served as the light propagation direction in our experiments. The β‐strands allows for an efficient growth of two very stable antiparallel β‐sheets in the *a*–*b* plane for each couple of strands (Figure 4c,d). Along the *c*‐axis, growth is governed by π‐interactions between adjacent aromatic rings (orange dashed lines between aromatic rings in Figure 4e). Surprisingly it seems that only a handful of π interactions exist along this axis, as compared to a multitude of stronger hydrogen bonds along the *a* and *b* axes. A view along the *a*‐axis reveals channels of ≈11×5 Å (black rectangle Figure 4e,f), which are large enough to host small molecules such as water and methanol as well as smaller entities such as metallic ions.^27^ The water inside the channels form 2D‐like sheets (red dots in Figure 4f), which are hydrogen bonded to the two β‐sheets, and are probably essential in order to maintain the structure given the otherwise weak interaction between the sheets. The inner walls of these channels consist of both hydrophilic (charged amine and carboxyl) as well as hydrophobic groups (aromatic rings), which differentiates them from the fully hydrophilic channels of FF‐tubes.^42^ The porous structure with extensive antiparallel β‐sheets we discovered confirms previous predictions based on molecular dynamics simulations for FFF self‐assembly.^43^, ^44^

The origin of the electro‐optic response in the crystal structure is not observed at first sight. The large dipole moments of the zwitterions lie along the *b*‐axis, but the molecules themselves are arranged in an almost perfect antiparallel arrangement such that the net dipole is but a small fraction. Along the *a*‐axis, there are intramolecular contributions from the amide dipoles, but these as well are mostly cancelled due to the antiparallel nature. However, l‐phenylalanine is a left handed chiral molecule, and therefore all its homopeptides and derivatives must crystalize in a noncentrosymmetric space group.^45^ This property of chiral amino acids was used before in order to engineer materials with strong nonlinear optical response.^46^, ^47^, ^48^ The triclinic P1 space group of the tapes (Table S1, Supporting Information) is indeed noncentrosymmetric, and it is actually the lowest possible symmetry group, therefore some contribution from each dipole remains, which eventually adds up to the response we observed. It is nevertheless delicate to assign a precise structural origin to the response we measured. The situation is very different in the FF tubes. The FF tube is comprised by many parallel nanotubes,^42^ that are arranged in a hexagonal packing (Figure S5, Supporting Information), such that their hydrophilic charged groups are all exposed to the center of the tube where water resides, while the hydrophobic aromatic residues points toward each other (Figure S5b, Supporting Information). A closeup look along the nanotube's cross section shows that the sixfold symmetry cancels any dipolar contributions to the EO response in the *a*–*b* plane (black arrows indicate the strong dipole between the charged groups of the FF zwitterion, Figure S5c, Supporting Information). The picture changes dramatically along the tube wall. The FF molecules are arranged in a helix around the nanotube diameter.^40^ The zwitterion dipoles are aligned along the sixfold axis of the tube such that the dipolar contribution adds up in the long axis direction (Figure S5d, Supporting Information, and see also ref. 40) This better alignment leads to the much stronger response we observed in the tubes compared to the plates.

### 2.4 Observation of Domains in FFF‐Tapes

**Figure 5:**
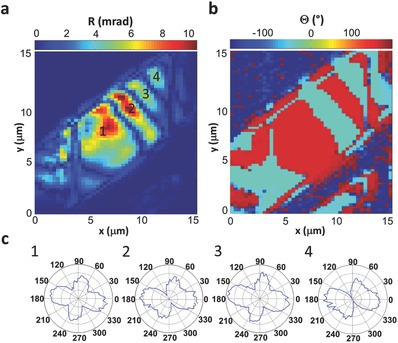
Multidomain tape. a) Retardance image of the tape. b) Θ image of the tape. c) Polar plots of different domains. Plots 1–4 correspond to the numbered domains in (b).

While some FFF‐tapes showed a homogenous electro‐optical response indicative of single domain structure as observed for the FFF‐tapes discussed above, other have shown heterogeneous distribution of intrinsic ordering of molecular dipoles. The PLEOM setup allows determination of the dipole orientation based on the polar plot as well as the Θ signal of the sample. A crystal with a single domain possesses a single polar plot and a single Θ‐value across it for every measured position along the crystal, while a single polar plot and alternating Θ values with 180° shift indicate domains alternating between dipole orientations such as seen in electrically poled ferroelectric crystals exhibiting inverted domains.^49^ A crystal with changing polar plots and varied Θ values is an indication of a more complex domain structure.^50^ An example of a multidomain structure is presented in **Figure** **5**. The FFF‐tape was imaged with polarization along the X‐axis of the laboratory frame as defined before (Figure 3b). The retardance image showed alternating stripes of high and low signals (Figure 5a). The corresponding Θ image (Figure 5b) showed alternating patterns with 180° shift, indicating variation of molecular dipole orientations. In order to uncover the individual domain structures, we measured the polarimetric response of four different domains (Figure 5c).

The change in both the polar plot and the Θ value is an indication that the domains are not organized in an antiparallel manner but display different orientations from one to the next. However, it seems that domains 1 and 3, and domains 2 and 4 share very similar structures, with similar polar plots and Θ‐value. The walls between the domains defined in the phase image (Figure 5b) showed a weaker response in the amplitude image (faint stripes in Figure 5a) which may be assigned to an overlap at the beam waist of domains with different polarity orientations, leading to an overall reduction of the electro‐optic signal, all the way to full cancellation. The domains polarization response showed a more complex behavior with respect to the single domain structures, which repeated itself in other multidomain tapes (see also Figure S6, Supporting Information). This complex response suggests that the arrangement in the multidomain structures might be rotated with respect to the single domain structures. However, this assumption requires further investigation by complementary methods, e.g., piezo force microscopy. Nevertheless, the conclusive results of the spontaneously formed multidomains with varying electro‐optic response in a single self‐assembled peptide structure, motivates the exploration of ways to engineer the domain pattern for future potential applications.

## 3 Conclusions

We have observed for the first time strong linear electro‐optic response in bio‐organic phenylalanine homopeptides microstructures. The electro‐optic coefficients for both assemblies have demonstrated very large values similar to that of commercially available inorganic electro‐optic crystals and in FF‐tubes reached ≈32 pm V^−1^ for *r*_33 _in line with the best ferroelectric crystals. XRD studies of FFF‐tapes have shown a porous structure of antiparallel β‐sheets capable of hosting guest entities such as water and ions as well as larger molecules. By measuring electro‐optic activity, we have revealed in FFF‐tapes a complex state of polarization and a pronounced structure for domains. The efficient optical nonlinearity of these structures, their porosity, biocompatibility and domain formation can lead to the establishment of new functional optical materials by the incorporation of guest molecules, novel chemical, and biological optical sensors, and new types of self‐assembled bioorganic electro‐optic modulators.

## 4 Experimental Section

*Peptide Self‐Assembly*: FF and FFF were purchased in lyophilized form from Bachem (Switzerland). The peptides were dissolved in 1,1,1,3,3,3‐hexafluoro‐2‐propanol at concentrations of 100 and 50 mg mL^−1^, respectively. The solutions were diluted in water to a final peptide concentration of 1.5 mg mL^−1^ each. For FF, a drop of 10 µL of fresh FF solution in water was left to dry on an electrode patterned glass coverslip to form tubes. FFF solution was first aged for 3 d until crystal aggregates were visible. A drop from the aged solution was gently placed on an electrode coated glass to prevent breakage due to the high aspect ratio of the tapes, while removing excess solution in order to minimize spontaneous self‐assembly on the slide. Both the FF and FFF solutions were optimized to produce the largest and best quality structures by controlling the concentrations, ratio of solvents, and growth temperature.

*Electrode Fabrication*: For electrode fabrication a 10 nm thick chromium layer was deposited on a standard microscope coverslip followed by 50 nm of gold. Standard lithography and etching techniques were then used to pattern the gold electrodes on the slide.

*PLEOM Setup*: The PLEOM setup was used as was described before.^34^ A He:Ne laser at 633 nm was split to two beams using a polarizing beam splitter (PBS) with a ratio of 9:1 between the intensities of the reference and signal beam, respectively. The signal arm passed a motorized half wave plate to control its incident polarization. The beam was then fed to a custom built confocal microscope made of two identical objectives (Plan Fluar 40×/0.6, Nikon). The gold electrode patterned coverslip was mounted on a piezoelectric stage (Piezosystem Jena) used to scan the sample. The signal beam acquired a phase retardance due to the Pockels effect induced by an AC field of 150 V at 60 kHz in the sample. The beam then passed another half wave plate, with the same orientation as the first, such that an unmodulated beam would return to its original polarization, and a polarizer at the same orientation.

The two beams were recombined using a PBS, and the interfering beam was then split and projected onto two photodiodes (Hamamatsu) in balanced homodyne detection configuration. The signal was fed to a Lock‐in amplifier (EG&G Princeton Applied Research) tuned to the modulation frequency. In the reference arm a mirror mounted on a piezoelectric stage (dubbed phase correcting unit (PCU) in Figure 2) was fed by a low frequency signal from the photodiodes, in order to alter the optical path of the reference beam such that a 90° phase separation would be maintained between the sample and reference arm, which provided the best interference signal.

A commercial pottasium dideuterium phosphate (KD*P) based Pockels cell (Leysop) was also stationed in the signal arm before the first half wave plate, and it was modulated alternately with the sample such that a known retardance was achieved. The signal from the Pockels cell was used to calibrate the retardance measured by the lock‐in amplifier.

*AFM Images*: The images were acquired on NanoWizard 3 BioScience AFM in tapping mode. Noise gain was set to 150 kHz, scan rate was 0.3 Hz. The tip used was HQ:NSC15/Al BS with ≈290 kHz resonance.

*Environmental SEM*: The samples were coated with palladium and scanned using a scanning electron microscope (JSM‐6300, JEOL) operating at 10–15 kV.

*Light Microscopy*: Bright field and dark field images were taken on a Zeiss Axio observer, equipped with a 10X/0.25 objective. Images were captured using a single‐lens reflex color camera (EOS 650D, Canon).

*Interferometric Microscopy*: Light from a supercontinuum laser (Fianium) was filtered using an acousto‐optic tunable filter (Crystal Technology) to transmit a semicoherent beam of 650 nm wavelength with 7 nm spectral width, which was then incident on the sample. A 60X/1.45 oil immersion objective (Olympus) was used to collect the transmitted light. A τ interferometer unit positioned after the objective created a reference signal by filtering the high order Fourier components of the image, and then interfered the resulting reference signal with the original image in an off axis configuration. The interference signal was recorded on a complementary metal oxide semiconductor (CMOS) camera (Thorlabs), and refractive index images were obtained by analysis in custom written MATLAB software (MathWorks).

*Single X‐Ray Diffraction*: The FFF crystal was transferred to Paratone oil (Hampton Research) and mounted on a MiTeGen loop. The crystal was flash frozen in liquid nitrogen.

Crystal data for the FFF‐tape was measured at 100 K on a Rigaku XtaLabPro diffractometer equipped with [λ (Cu*K*_α_) = 0.154184 Å] radiation and a Dectris Pilatus S200 detector, the data were processed with CrysAlisPro programs (Rigaku). The Structure was solved by direct methods with SHELXT‐2013 and refined with full‐matrix least squares refinement based on F2 with SHELXL‐2013.

[CCDC 1493588 contains the supplementary crystallographic data for this paper. These data can be obtained free of charge from The Cambridge Crystallographic Data Centre via www.ccdc.cam.ac.uk/data_request/cif.].

## Conflict of Interest

The authors declare no conflict of interest.

## Supporting information

Supplementary
