# Supplementary material for: Strong Electro‐Optic Effect and Spontaneous Domain Formation in Self‐Assembled Peptide Structures
Source: Adv Sci (Weinh). 2017 May 11;4(9):1700052. doi: 10.1002/advs.201700052 (PMC5604517; doi:10.1002/advs.201700052)
Supplement: Supplementary file 1 — Supplementary [file ADVS-4-na-s001.pdf]

Copyright WILEY-VCH Verlag GmbH & Co. KGaA, 69469 Weinheim, Germany, 2017.

Supporting Information

for *Adv. Sci.,* DOI: 10.1002/advs.201700052

Strong Electro-Optic Effect and Spontaneous Domain Formation in Self-Assembled Peptide Structures

Barak Gilboa, Clément Lafargue, Amir Handelman, Linda J. W. Shimon, Gil Rosenman, Joseph Zyss, and Tal Ellenbogen**

Supporting Information

Strong Electro Optic Effect and Spontaneous Domain Formation in Self Assembled Peptide Structures

Barak Gilboa, Clément Lafargue, Amir Handelman, Linda J.W. Shimon, Gil Rosenman, Joseph Zyss, Tal Ellenbogen**

Supporting Information

Strong Electro Optic Effect and Spontaneous Domain Formation in Self Assembled Peptide Structures

Barak Gilboa, Clément Lafargue, Amir Handelman, Linda J.W. Shimon, Gil Rosenman, Joseph Zyss, Tal Ellenbogen**

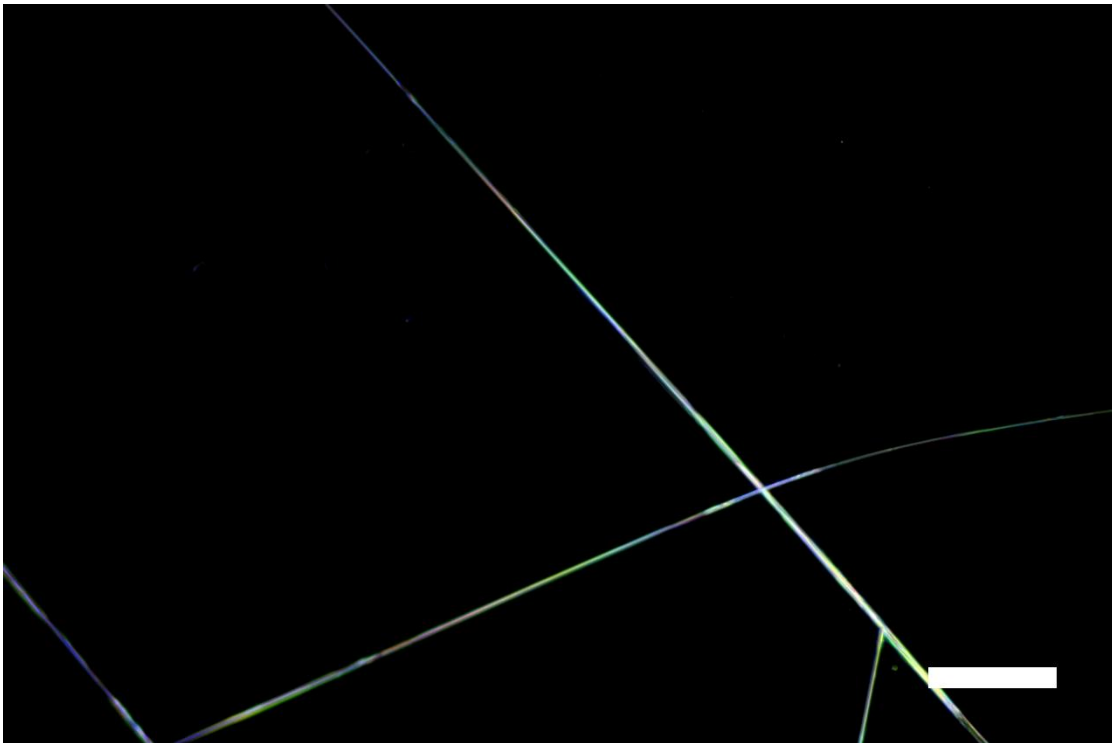

Figure S1. Dark field image of FF-tubes. Scale bar is 100 μm.

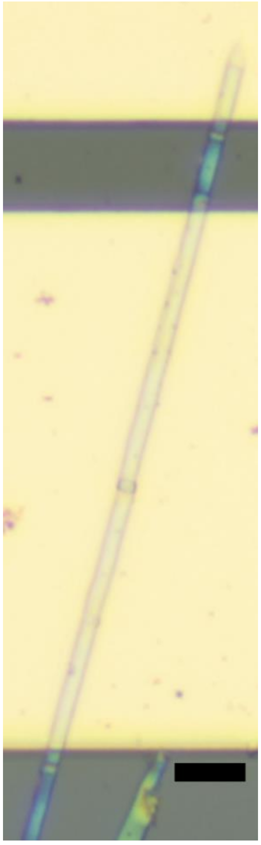

Figure S2. FFF-tape on top of gold electrodes. Electrodes are yellow. Scale bar is 5 μm.

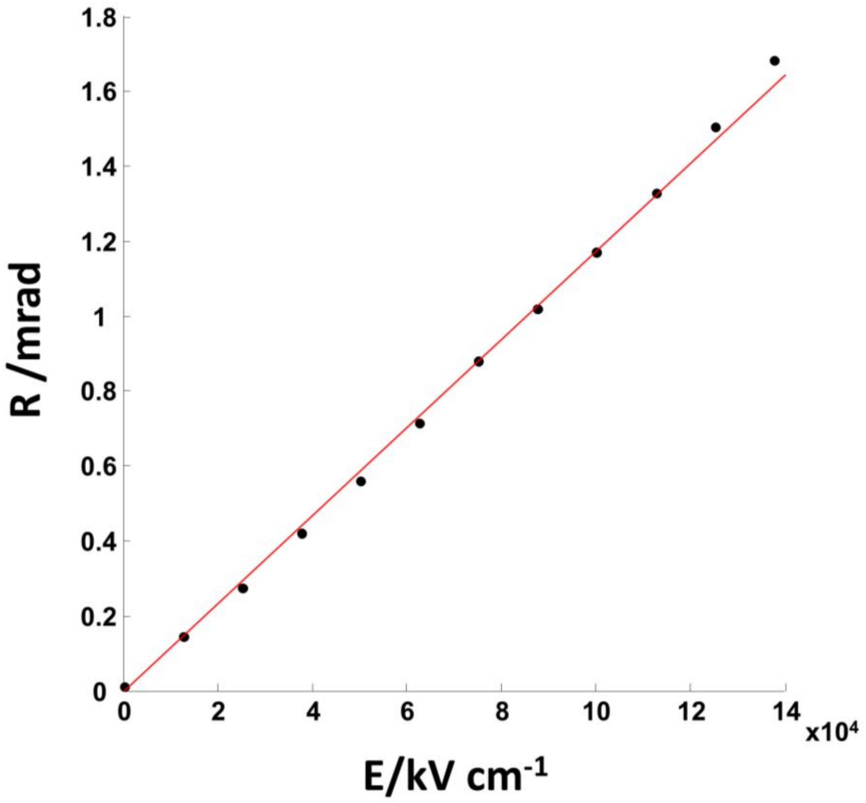

Figure S3. Plot of retardance vs. applied electric field. The red line is a linear fit to the data.

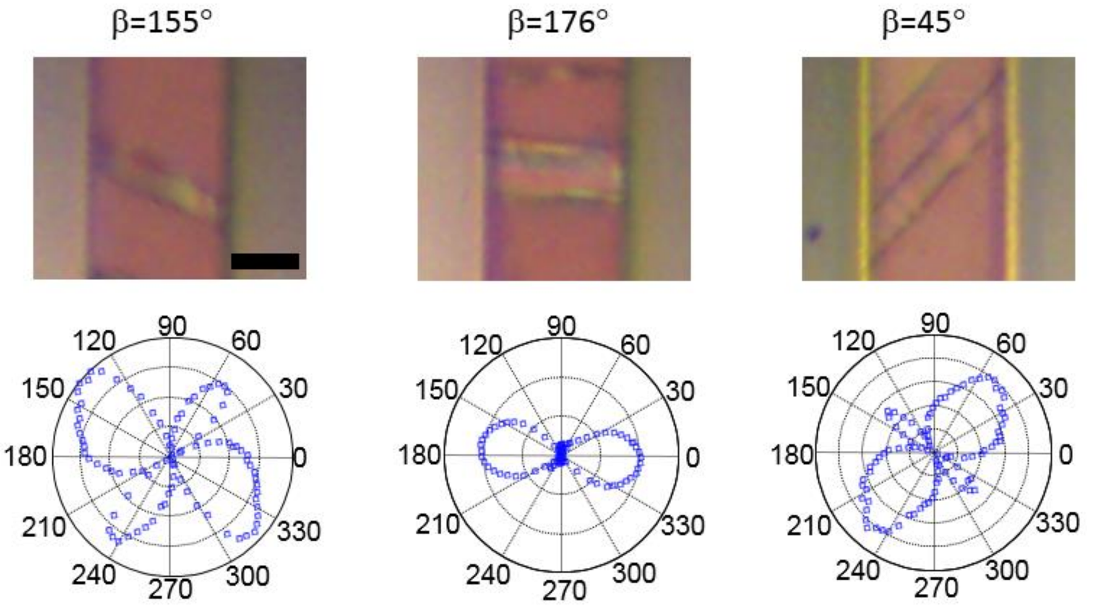

Figure S4. Single domain FFF-tapes with differing orientations**.** Top: Bright field images of the tapes. β denotes the angle from the electrodes to the tape. Bottom: the corresponding polar plots of the same tapes. Bar size is 5 µm.

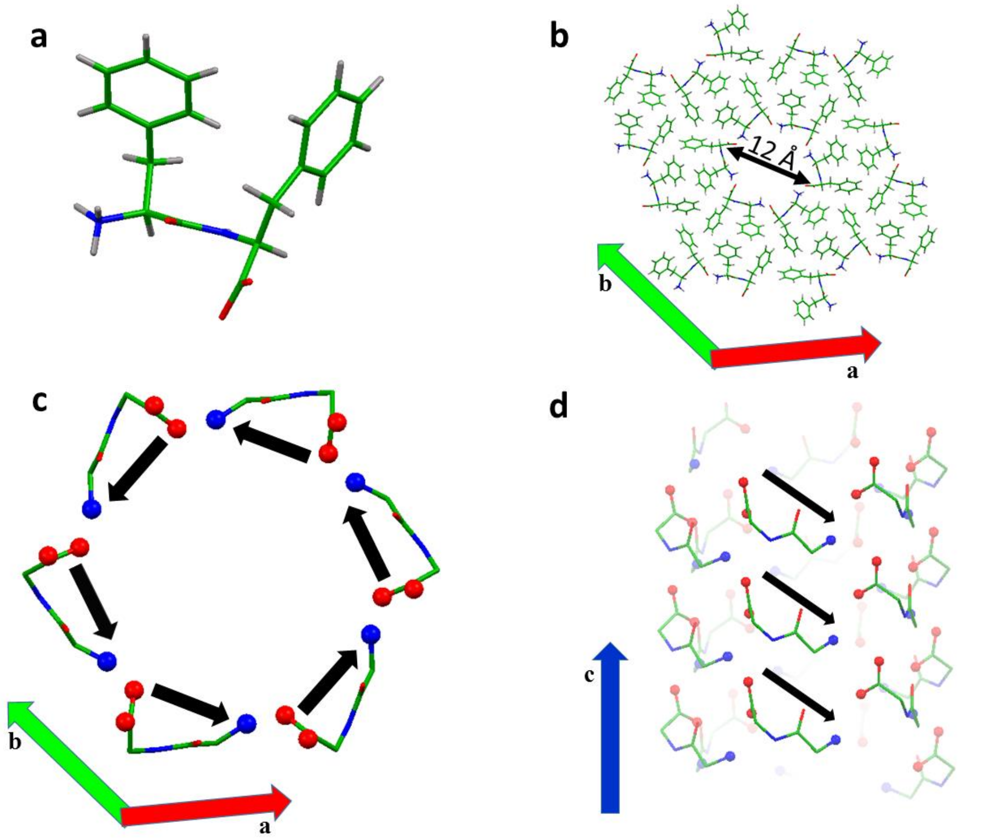

Figure S5. FF structure and dipolar contributions to EO response. **a.** FF molecule in its zwitterionic form. Carbons are green, oxygens in red and nitrogens in blue. **b.** A cross section

of a nanotube within the macroscopic tube structure. a and b are the crystallographic axes. **c.** A close up on the backbone of the nanotube inner wall. The black arrows denote the strong dipole of the charged end groups in the zwitterion. Hydrogens and aromatic side chains were omitted for clarity. Red and blue balls mark the oxygens and nitrogens of the charged end groups. **d.** The backbone of FF molecules comprising the tubular helix of a nanotube.

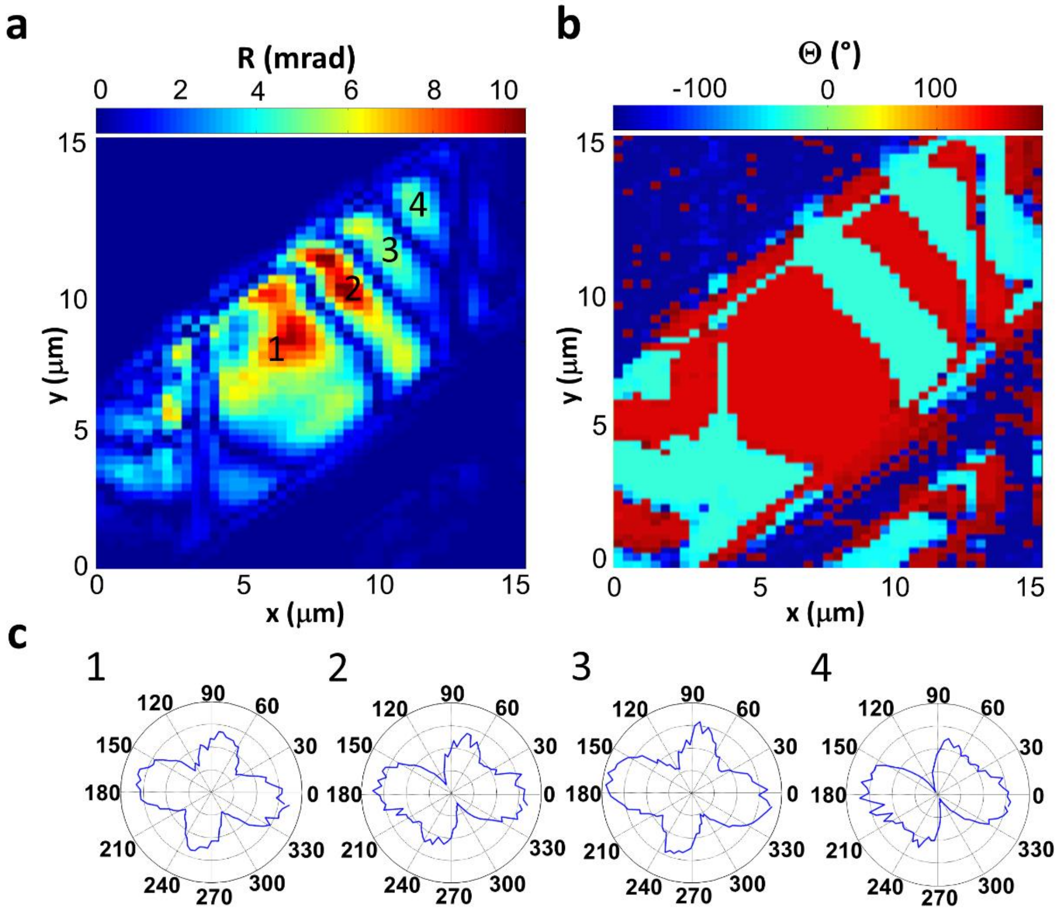

Figure S6. Multi-domain structure of a tape. a) PLEOM retardance image. b) PLEOM Θ image. (c-d) Zoomed in retardance and Θ images of the area marked by a rectangle in b. e) Polar plots of the different domains in c-d.

Supplementary note 1: Estimation of FF thickness

In order to estimate the effective thickness of FF, we will look at the cross section of the tube as a ring made of two concentric circles. This is a fair approximation given the hexagonal shape of the tube. The maximal thickness of such a ring is achieved when the distance from the edge is exactly the difference between the outer and inner radii of the ring. Since the electro-optic response is linearly dependent on the thickness, we looked at the cross section

of the tube and found the maximum value is achieved at a distance of ~0.9 μm from the tube edge. The thickness at a given distance from the edge is described by:

$$d = \sqrt{(2R - x)x}$$

Where R is the radius and x is the distance from the edge. Given the radius of the analyzed tube is 1.25 μm, the corresponding thickness is $d = 1.2$ μm.

Supplementary note 2: FF coefficients calculation

In order to calculate the exact coefficients we must know the space group of the crystal. For the FF-tubes it is $P6_1$, and therefore In the crystal frame (X, Y, Z) the electro-optic tensor is :

$$r = \begin{pmatrix} 0 & 0 & r_{13} \\ 0 & 0 & r_{13} \\ 0 & 0 & r_{33} \\ -r_{41} & r_{51} & 0 \\ r_{51} & r_{41} & 0 \\ 0 & 0 & 0 \end{pmatrix} \quad (1)$$

in this frame the external electric field applied along y is: $E = \begin{pmatrix} E\sin(\beta) \\ 0 \\ E\cos(\beta) \end{pmatrix}_{(X,Y,Z)}$

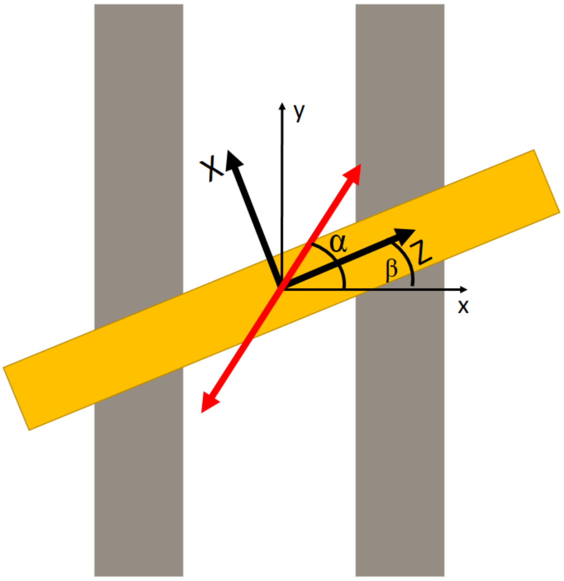

Figure S6. Schematic diagram of an FF tube in PLEOM and corresponding electric fields**.** (x,y) denotes the laboratory frame work, where x is the direction of applied AC electric field.

(X,Z) denotes the tube's framework. The double headed red arrow is the axis of the incident polarization, where α is the angle between the polarization and the x axis, and β is the angle between x and the long axis of the tube (Z).

Then: $$\Delta\left(\frac{1}{n^2}\right) = \begin{pmatrix} 0 & 0 & r_{13} \\ 0 & 0 & r_{13} \\ 0 & 0 & r_{33} \\ -r_{41} & r_{51} & 0 \\ r_{51} & r_{41} & 0 \\ 0 & 0 & 0 \end{pmatrix} . \begin{pmatrix} E\sin(\beta) \\ 0 \\ E\cos(\beta) \end{pmatrix} \quad (2)$$

Then if we assume a uniaxial crystal, based on the space group symmetry, we can call: $\frac{1}{n'^2_0} = \frac{1}{n_o^2} + r_{13}E\cos($[illegible] $\frac{1}{n'^2_e} = \frac{1}{n_e^2} + r_{33}E\cos($[illegible]

The index ellipsoid then becomes :

$$\frac{X^2}{n'^2_0} + \frac{Y^2}{n'^2_0} + \frac{Z^2}{n'^2_e} + (2YZr_{41} + 2XZr_{51})E\cos(\beta) = 1 \quad (3)$$

We will for now omit the YZ contribution in our calculation, and focus on the XZ term in order to demonstrate that cross terms effects are very small. The cross terms rotate the refractrive index ellipsoid. We therefore would like to return to an equation of the form: $\frac{x^2}{n_x^2} + \frac{y^2}{n_y^2} + \frac{z^2}{n_z^2} = 1$, by rotating the axes by an angle θ:

$$\begin{aligned} Y &= y \\ X &= x\cos\theta - z\sin\theta \\ Z &= x\sin\theta + z\cos\theta \end{aligned}$$

With θ defined by: $$\tan 2\theta = \frac{2r_{51}E\cos(\beta)}{\frac{1}{n'^2_0} - \frac{1}{n'^2_e}} \quad (4)$$

so we have the final indices :

$$\begin{cases} n_x = n'_0 + \frac{1}{2} n'^3_0 r_{51} E\cos(\beta)\tan(\theta) \\ n_y = n'_0 \\ n_z = n'_e - \frac{1}{2} n'^3_e r_{51} E\cos(\beta)\tan(\theta) \end{cases} \quad (5)$$

From Equation (4) it can be seen that the effect of the cross term is strongly dependent on the birefringence, $\frac{1}{n{\prime}_0^2} - \frac{1}{n{\prime}_e^2}$, of the material, since the numerator is very small relative to the denominator even for mild birefringence, as we observed for FFF-plates, and high values of the electro-optic coefficient and the applied field. While the refractive indices of FF are unknown to date, we can estimate the response using the values for FFF-plates. using FFF's birefirngence - $\frac{1}{n{\prime}_0^2} - \frac{1}{n{\prime}_e^2} = 0.02$, the applied field - $E = 1.5 \cdot 10^5 \frac{V}{cm}$, and taking the value of $r_{51}$of $LiNBO_3$ - $r_{51} \cong 32\ \frac{pm}{V}$,among the higest cross term coefficients measured, yields $\tan(\theta) \cong 0.03$, corresponding to a rotation of just 1.4°, and its electro-optic contribution is negligible. Therefore, we will only conisder the contributions of r_{13}, r_{33} to the electro-optic response from here on.

<u>The signal in PLEOM for FF-tubes</u>

The incident field is the following, in (x,y,z) and (X,Y,Z) frameworks respectively :

$$E^{\omega}\begin{pmatrix}\cos\alpha \\ \sin\alpha \\ 0\end{pmatrix}_{x,y,z} = E^{\omega}\begin{pmatrix}\sin(\alpha-\beta) \\ 0 \\ \cos(\alpha-\beta)\end{pmatrix}_{X,Y,Z} \qquad (8)$$

After going through the tube , the field is

$$E_s = E^{\omega}\begin{pmatrix}\sin(\alpha-\beta)e^{ikn_X d} \\ 0 \\ \cos(\alpha-\beta)e^{ikn_Z d}\end{pmatrix}_{X,Y,Z} \qquad (9)$$

Where k is the wave number of the incident laser beam, and d is the effective thickness calculated above. The beam then goes through a halfwaveplate at $\alpha/2$ and a polarizer along x

which is equivalent to projecting this field along $\vec{e}_\alpha$, the vector aligned with the incoming polarization:

$$\underline{E_s} = E^\omega\left[\sin^2(\alpha-\beta)e^{ikn_X d} + \cos^2(\alpha-\beta)e^{ikn_Z d}\right] \tag{10}$$

This can be written as the sample beam : $\underline{E_s} = E_S e^{i\phi_S}$ with a modulus E_S and a phase ϕ_S that will be detailed hereafter.

Under the influence of the quasistatic Electric field applied by the electrodes $E^\Omega \vec{e}_y$, the indexes are modified.

$$n_X \rightarrow n_0 + \frac{n_0^3}{2} r_{13} E^\Omega \cos(\beta) \tag{11}$$

$$n_Z \rightarrow n_e + \frac{n_e^3}{2} r_{33} E^\Omega \cos(\beta) \tag{12}$$

So that the modulus is $E_s \rightarrow E_s + \delta E_s$, with a phase $\phi_S \rightarrow \phi_s + \delta\phi_S$. By mixing it with a well chosen reference beam of the form: $E_r e^{i\phi_S + \pi/2}$, The total field and the intensity associated become:

$$E_t = E_s e^{i(\phi_S + \delta\phi_s)} + E_r e^{i(\phi_S + \frac{\pi}{2})} \tag{13}$$

$$I_t = |E_t|^2 = E_s^2 + E_r^2 + 2E_s E_r \sin(\delta\phi_s) \tag{14}$$

The balanced homodyne detection of the signal allows us direct access to the intererence term:

$$\delta I = 2E_s E_r \sin(\delta\phi_s) \cong 2E_s E_r \delta\phi_s \, , \delta\phi_s \ll 1 \tag{15}$$

The signal depends on E_s and ϕ_s that both depend on E^Ω as detailed hereafter.

<u>The modulus</u>

The modulus of the sample beam is:

$$E_s(\alpha) = E^{\omega}\sqrt{\sin^4(\alpha-\beta) + \cos^4(\alpha-\beta) + 2\sin^2(\alpha-\beta)\cos^2(\alpha-\beta)\cos(kd(n_X - n_Z))} \quad (16)$$

assuming $n_Z = 1.6$, $n_X = 1.55$, $d = 1.2\ \mu$m and $\lambda = 2\pi/k = 632$ nm, then $kd(n_X - n_Z) \simeq 0.6$, which means E_s is a function that varies periodically with α to the tune of at most <11%.

<u>The phase</u>

the phase of $\underline{E_s}$ is defined by:

$$\phi_s = \arctan\left(\frac{Ims}{Res}\right) \quad (17)$$

where Ims and Res are the imaginary and real parts of $\underline{E_s}$.

$$Ims/E^{\omega} = \sin(\alpha-\beta)^2\sin(kn_Xd) + \cos(\alpha-\beta)^2\sin(kn_Zd) \quad (18)$$

$$Res/E^{\omega} = \sin(\alpha-\beta)^2\cos(kn_Xd) + \cos(\alpha-\beta)^2\cos(kn_Zd) \quad (19)$$

We will consider the change of ϕ_s with E^{Ω}:

$$\frac{d\phi_s}{dE^{\Omega}} = \frac{Ims'Res - ImsRes'}{Res^2 + Ims^2} \quad (20)$$

$$\frac{\delta\phi_s}{dE^{\Omega}} = kd\,\frac{n'_X\sin(\alpha-\beta)^4 + n'_Z\cos(\alpha-\beta)^4 + \sin(\alpha-\beta)^2\cos(\alpha-\beta)^2\cos(kd(n_X-n_Z))(n'_X + n'_Z)}{(E_s/E^{\omega})^2} \quad (21)$$

$$\delta\phi_s = kd\,\frac{\delta n_X\sin(\alpha-\beta)^4 + \delta n_Z\cos(\alpha-\beta)^4 + \sin(\alpha-\beta)^2\cos(\alpha-\beta)^2\cos(kd(n_X-n_Z))(\delta n_X + \delta n_Z)}{(E_s/E^{\omega})^2} \quad (22)$$

The lock in amplifier is tuned to the frequency Ω of the applied external field, and therefore its signal is directly proportional to $\delta\phi_s$. From Equation (22) it is evident that no signal is measured when there is no electro-optic response in the material. Fitting Equation (22) to the polar plot, and comparing it to the measured retardance by the pockels cell, allows the

determination of δn_X, δn_Z which allows the caclulation of the respective electro-optic coefficients.

Supplementary table 1: Crystal structure data for FFF tapes

Complex	**FFF-tape**
CCDC Deposition #	1493588
Formula	$C_{27}H_{29}N\ O_4$, 3(O)
Crystal description	colourless plate
Crystal size, [mm^3]	0.20 x 0.08 x 0.01
FW, [$g.mol^{-1}$]	507.53
Space group	*P1*
Crystal system	Triclinic
a, [Å]<br>b, [Å]<br>c, [Å]<br>α, [°]<br>β, [°]<br>γ, [°]	9.7001(1)<br>12.878(1)<br>22.443(2)<br>96.416(8)<br>94.178(10)<br>90.466(9)
Cell volume, [$Å^3$]	2778.3(5)
Z	4
ρ_{cacld}, [$g.cm^{-3}$]	1.213
μ, [mm^{-1}]	0.73
No. of reflections	14611
No. of unique reflections	6864
$2\Theta_{max}$, [°]	88.96
R_{int}	0.116
No. of parameters (restraints)	1198(1223)
Final R^a	0.1562
Final R^b	0.1954
GooF	1.431

[a] for data with $I > 2\sigma(I)$. [b] for all data.
